# Headache as an extraocular manifestation of birdshot chorioretinopathy: a case series

**DOI:** 10.3389/fopht.2026.1838200

**Published:** 2026-08-13

**Authors:** Jonathan D. Groothoff, Maninder K. Sohi, Hannah Shuman, Brandy W. Hardy, Robert C. Goodrich, Kristyn S. Pocock

**Affiliations:** 1 Wake Forest University School of Medicine, Winston-Salem, NC, United States; 2 Department of Neurology, Wake Forest University School of Medicine, Winston-Salem, NC, United States; 3 Department of Ophthalmology, Wake Forest University School of Medicine, Winston-Salem, NC, United States; 4 Comprehensive Headache Program, Atrium Health Wake Forest Baptist, Winston-Salem, NC, United States

**Keywords:** birdshot, birdshot chorioretinopathy, case series, headache, HLA-A29

## Abstract

Birdshot chorioretinopathy (BSCR) is a type of chronic posterior uveitis with limited characterization of extraocular manifestations, including headache. We describe two HLA-A29-positive patients with BSCR who developed refractory unilateral periorbital headaches temporally associated with visual symptom onset. Headaches lateralized to the more affected eye and the patients exhibited migrainous and prominent cranial autonomic features resembling trigeminal autonomic cephalalgias (TACs) and variable responses to standard headache therapies. Together, this supports the International Classification of Headache Disorders (ICHD-3) classification as a secondary headache attributed to ocular inflammatory disease. The biological plausibility of this association is supported by trigeminal innervation of the uveal tract, providing a substrate for inflammation-driven trigeminovascular activation. Systematic studies are needed to define BSCR-associated headache prevalence, phenotype, and mechanisms and to inform diagnosis and management.

## Introduction

Birdshot chorioretinopathy (BSCR) is a rare bilateral posterior uveitis with a prevalence of 0.1 to 0.6 cases per 100,000 (1). The pathophysiology is incompletely understood, although a strong association with HLA-A29 haplotype positivity exists (1). Common visual complaints include gradual vision loss, floaters, photopsia, and color and night vision changes, with characteristic hypopigmented choroidal lesions appearing similar to “birdshot” ammunition fired upon a target visible on imaging. BSCR is largely considered an ocular disorder with few extraocular manifestations described (2). Previous studies have reported coexistent hypertension, tinnitus, hearing loss, arthralgia, and psoriasis, although no predominant extraocular manifestations have been identified (2–4). We present two cases of patients with BSCR who presented with visual changes and headache with atypical features. Our aims were 1) to determine whether BSCR has been described with headache and 2) to characterize headache features in those affected. To the best of our knowledge, this is the first study to describe in detail a temporal association between BSCR-related visual symptoms and the onset of intractable headache with trigeminal autonomic cephalalgia features, which we further define using International Classification of Headache Disorders (version 3) (ICHD-3) criteria.

## Case information

### Patient 1

**Figure 1:**
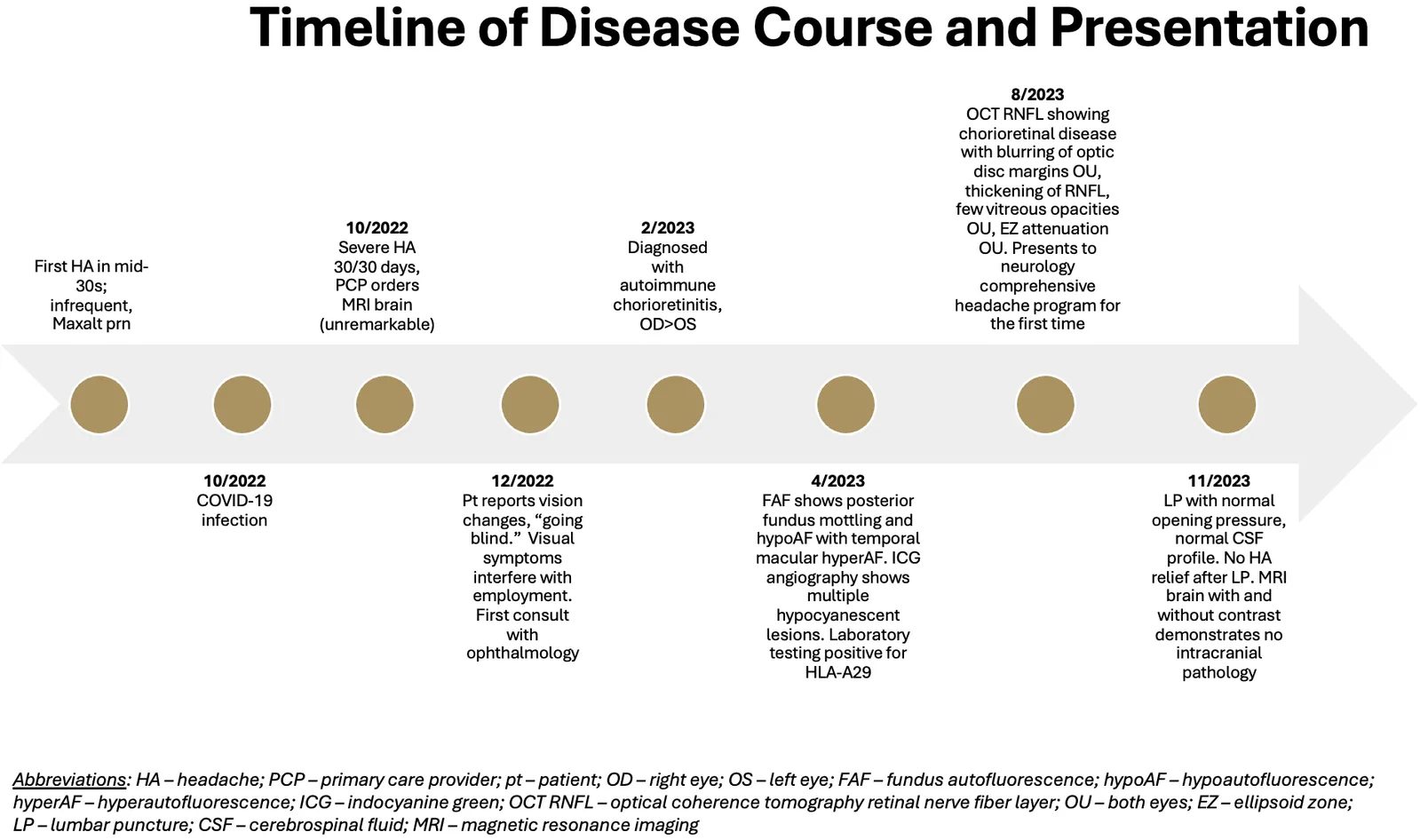
Timeline of patient disease course prior to diagnosis and presentation at a comprehensive headache program.

**Figure 2:**
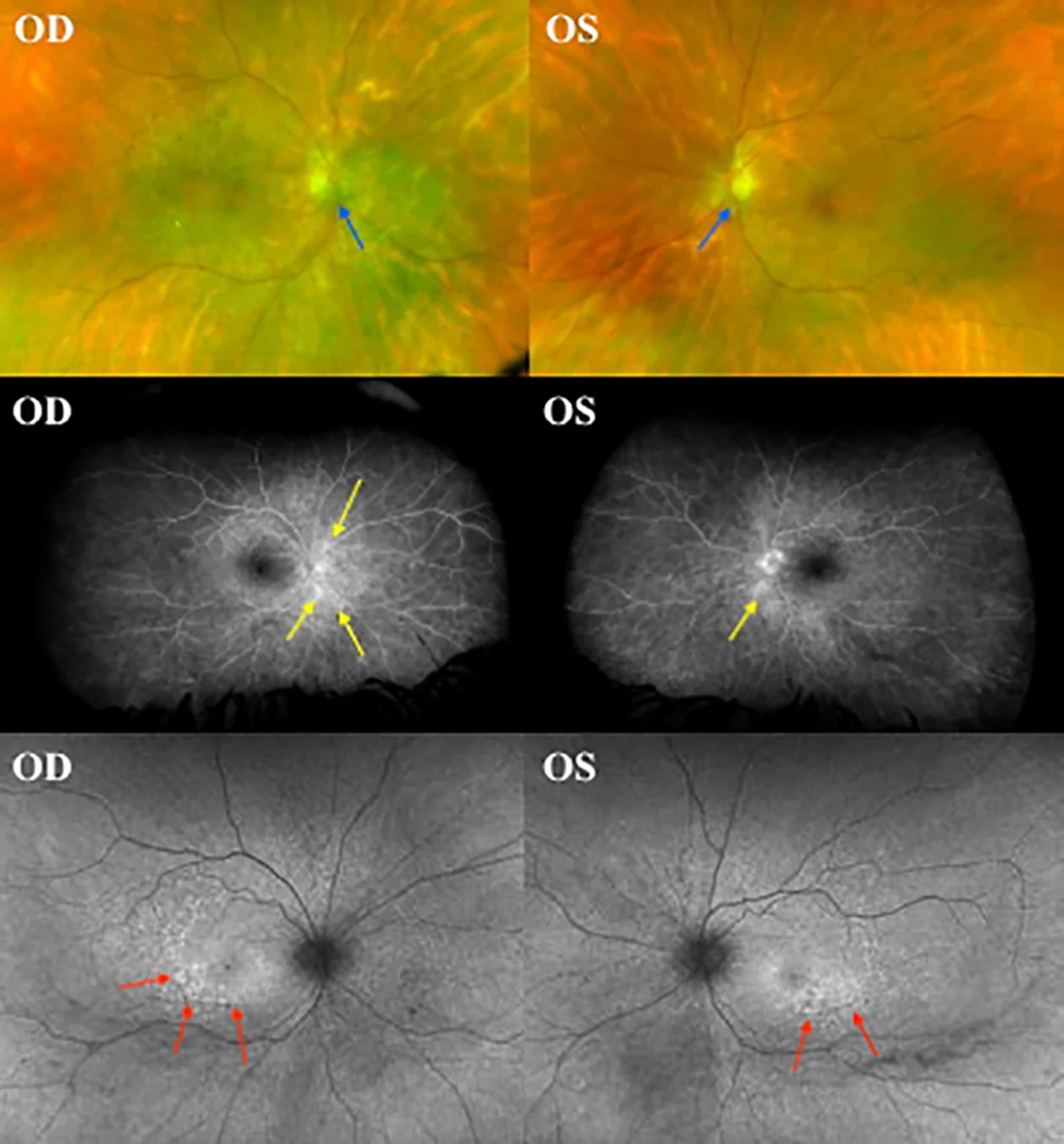
Imaging of Patient 1. Optos fundus photographs (top) revealed yellow retinochoroidal lesions radiating away from the disc in a scattershot pattern and mild disc edema bilaterally (blue arrows). Fluorescein angiography (middle) demonstrated perivascular and optic disc leakage, right eye worse than left (yellow arrows). Fundus autofluorescence (bottom) revealed bilateral mottled hypoautofluorescence and granular hyperautofluorescent lesions around the macula (red arrows), indicating lipofuscin buildup and active inflammation of the retinal pigment epithelium.

**Figure 3:**
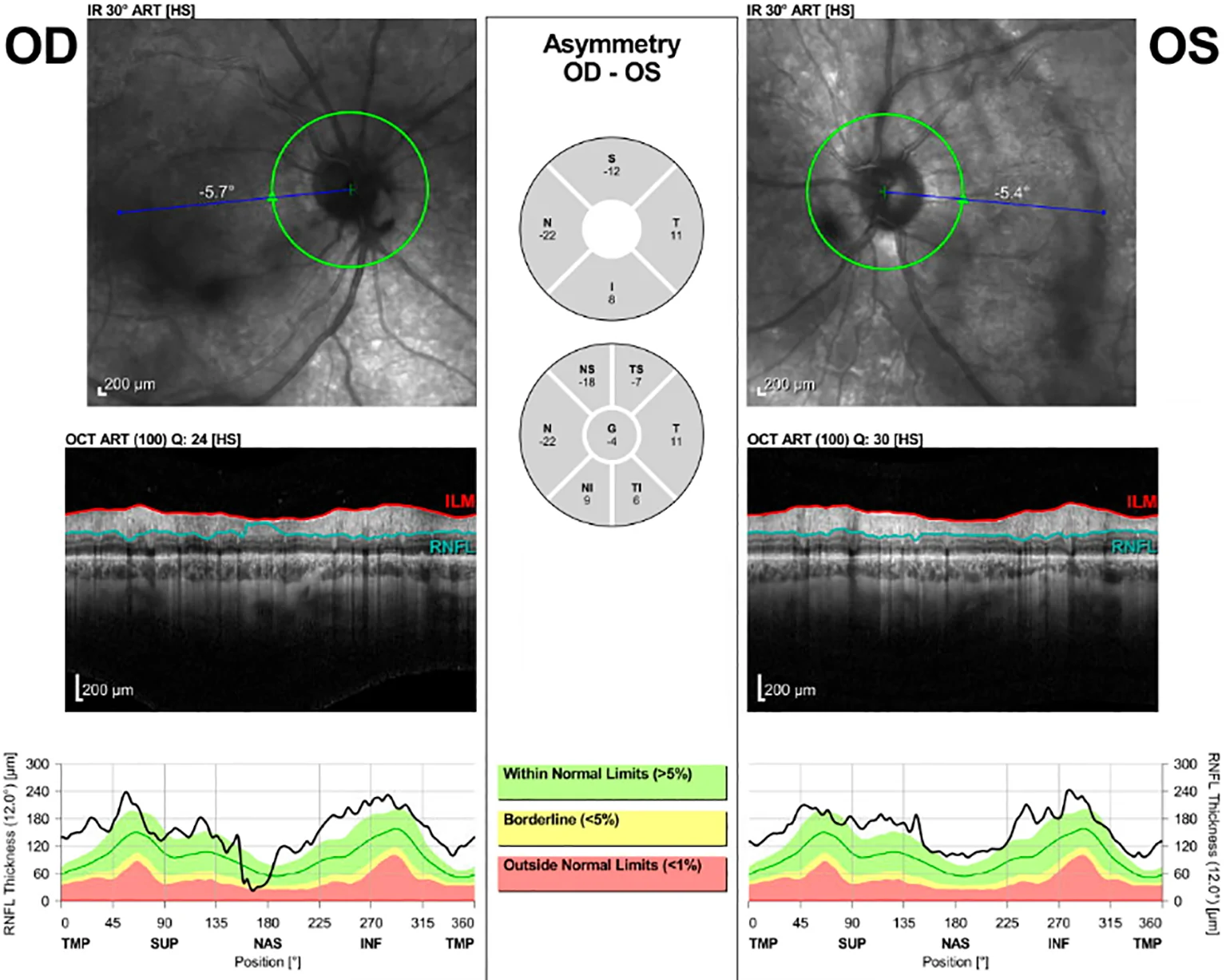
Optical coherence tomography (OCT) of the retinal nerve fiber layer (RNFL) of Patient 1. Thickening of the RNFL is apparent bilaterally.

A 43-year-old woman with episodic migraine and no past ocular history presented to ophthalmology with diplopia, photopsia, and floaters 1 week after a COVID-19 infection (**Figure 1**). She reported feeling like she “was going blind”. Two months prior to the onset of visual symptoms, her headaches became constant with daily attacks of right periorbital pain ipsilateral to the more symptomatic eye. Ophthalmology noted an uncorrected visual acuity of 20/80 [right eye (OD)] and 20/50 [left eye (OS)] with bilateral (OU) optic nerve engorgement and nasal creamy-white retinal lesions on fundoscopy, OD worse than OS (**Figure 2**). Optical coherence tomography (OCT) demonstrated vitreous opacities and chorioretinal disease with retinal nerve fiber layer thickening (**Figure 3**). Laboratory testing revealed HLA-A29 positivity, supporting a BSCR diagnosis. She started prednisone and mycophenolate mofetil for treatment.

The patient subsequently established care with our comprehensive headache program. Prior to the onset of her visual symptoms, her headaches satisfied the ICHD-3 criteria for episodic migraine. With her new headache phenotype, she reported severe right-sided supraorbital pain attacks lasting up to 3 hours without treatment, accompanied by photophobia, phonophobia, and nausea. She described autonomic features including right-sided aural fullness, ptosis, lacrimation, rhinorrhea, and congestion with alarm clock periodicity of her headaches at 1:00 AM, 3:00 AM, and 5:00 PM, features and symptoms often associated with a cluster headache phenotype (**Table 1**). She also reported intermittent left face and arm numbness and paresthesias with severe headaches lasting 30–60 minutes. Contrast magnetic resonance imaging (MRI) of the brain and computed tomography angiography (CTA) of the head and neck were normal. Lumbar puncture showed a normal opening pressure (16 cm H_2_O) and normal cerebrospinal fluid profile. The patient trialed numerous preventive and abortive/rescue headache medications and interventions (see **Supplementary Table 1** for a full list). She ultimately reported a meaningful reduction in headache intensity on a regimen of cluster-dose galcanezumab (300 mg monthly), candesartan, tizanidine, and onabotulinumtoxinA, with as-needed use of high-flow oxygen, although headache frequency remained unchanged. She was unable to tolerate verapamil due to hypotension, and she declined the use of lithium out of concern for adverse effects.

**Table 1:** International classification of headache disorders-3 (ICHD-3) diagnostic criteria for cluster headache, hemicrania continua, and headache attributed to an ocular inflammatory disorder.

Classification	Diagnostic Criteria
3.1 Cluster headache	A. At least five attacks fulfilling criteria B-D B. Severe or very severe unilateral orbital, supraorbital, and/or temporal pain lasting 15-180 minutes (when untreated) C. Either or both of the following: 1. at least one of the following symptoms or signs, ipsilateral to the headache: - conjunctival injection and/or lacrimation - nasal congestion and/or rhinorrhea - eyelid oedema - forehead and facial sweating - miosis and/or ptosis 2. a sense of restlessness or agitation D. Occurring with a frequency between one every other day and 8 per day E. Not better accounted for by another ICHD-3 diagnosis.
3.4 Hemicrania continua	A. Unilateral headache fulfilling criteria B-D B. Present for >3 months, with exacerbations of moderate or greater intensity C. Either or both of the following: 1. at least one of the following symptoms or signs, ipsilateral to the headache: - conjunctival injection and/or lacrimation - nasal congestion and/or rhinorrhea - eyelid oedema - forehead and facial sweating - miosis and/or ptosis 2. a sense of restlessness or agitation, or aggravation of the pain by movement - Responds absolutely to therapeutic doses of indomethacin - Not better accounted for by another ICHD-3 diagnosis
11.3.3 Headache attributed to ocular inflammatory disorder	A. Periorbital headache and eye pain fulfilling criterion C B. Clinical, laboratory and/or imaging evidence of an ocular inflammatory disease known to be able to cause headache C. Evidence of causation demonstrated by at least two of the following: 1. headache has developed in temporal relation to the onset of the ocular disorder 2. either or both of the following: - headache has significantly worsened in parallel with worsening of the ocular inflammatory disease - headache has significantly improved or resolved in parallel with improvement in or resolution of the ocular inflammatory disease 3. either or both of the following: - headache significantly improves with topical application of local anaesthetic agent to the eye - headache is aggravated by pressure applied to the eye 4. in the case of a unilateral ocular inflammatory disease, headache is localized and ipsilateral to it D. Not better accounted for by another ICHD-3 diagnosis.

### Patient 2

The patient’s 73-year-old mother (who had a remote history of episodic migraine) subsequently presented to our headache clinic. She described constant, intractable left periorbital pain, photophobia, phonophobia, and shooting pain in the left V1 and V2 distributions, with ipsilateral lacrimation, congestion, and conjunctival injection, symptoms associated with an ICHD-3 hemicrania continua phenotype (**Table 1**). She was diagnosed with BSCR shortly after her daughter, in the setting of gradual vision loss, “smoke-like” visual disturbances, and scintillating scotomas. She tested positive for HLA-A29 and had choroidal lesions classic for BSCR, OS more severe than OD. Best corrected visual acuity was 20/30 in both eyes. She started prednisone and adalimumab, reporting a 75% improvement in visual symptoms. She started nortriptyline and gabapentin (of which she felt only gabapentin was helpful) prior to establishing care at our comprehensive headache program. She was prescribed a trial of indomethacin (the treatment for hemicrania continua), and the patient reported resolution of headache at 25 mg three times daily (scheduled) dosing, which she trialed for 2 weeks; however, she developed gastrointestinal side effects despite prophylactic pantoprazole use. Indomethacin was, therefore, continued on an as-needed basis only. Interestingly, she admitted to trialing Patient 1’s suzetrigine (which was ineffective for Patient 1) and reported 24 hours of headache relief following each dose. However, Patient 2 was unable to obtain this agent through her own insurance. She continues to take scheduled gabapentin, indomethacin on an as needed basis, and is presently scheduled to begin treatment with onabotulinumtoxinA (a treatment she requested in light of her daughter’s improvement on this regimen.

## Discussion

Our study adds to a growing body of evidence that BSCR can present with headache, although current literature is limited. Pagnoux et al. (3) described a single-center analysis of 118 patients with BSCR who reported extraocular manifestations and found that 8.5% of patients experienced some non-specific “headache”, although the authors acknowledged that no predominant extraocular manifestation of BSCR was identified. Patel et al. (2) described a case of a patient with a remote history of “migraine” but noted that the patient denied headache at the time of BSCR diagnosis. Another case report described an Asian patient with BSCR who experienced non-specific “frequent headaches” with photophobia and reported an onset simultaneous to visual symptoms (4). To the best of our knowledge, no previous studies have clarified headache details using formal ICHD-3 criteria, and only one reported a temporal association between visual symptoms and headaches (4). Furthermore, a shared underlying mechanism linking common extraocular manifestations with BSCR pathophysiology has not been proposed or described.

In our patients, the temporal evolution, laterality, and treatment refractoriness of the headache disorders support a potential pathophysiological link to underlying ocular inflammation rather than a coincidental primary headache disease. Specifically, headaches were strictly or predominantly ipsilateral to the more affected eye, merged or transformed in parallel with ocular symptoms, and exhibited limited response to standard migraine-directed therapies (although Patient 2 has only returned for follow-up once to assess responsiveness), features that argue against an independent primary headache syndrome.

Although the clinical phenotypes included migraine features (photophobia, phonophobia, and nausea) and cranial autonomic symptoms resembling trigeminal autonomic cephalalgias (TACs), these findings are better interpreted as potentially reflecting shared downstream trigeminovascular activation rather than indicating a primary TAC diagnosis. Notably, both patients fail to meet ICHD-3 criterion E, “(headache) not better accounted for by another diagnosis” (**Table 1**), and the presence of active intraocular inflammation provides a biologically plausible alternative explanation. Accordingly, classification as *headache attributed to an ocular inflammatory disorder* is more appropriate than forcing categorization within primary headache syndromes such as cluster headache or hemicrania continua (**Table 1**). Regarding ICHD-3 11.3.3 criterion 3, tetracaine hydrochloride ophthalmic solution 0.5% was applied to the orbits of Patient 1 bilaterally to determine if there was any clinical improvement with anterior orbital anesthesia; however, this solution paradoxically worsened her pain. It should be noted, however, that this criterion is imperfect for disorders such as BSCR, which involve a posterior orbital distribution of inflammatory pathology and, thus, the utility of the anterior application of topical anesthetic is limited. The patient also endorsed significant worsening of her pain with application of orbital pressure.

The biologic plausibility of BSCR-associated headache is supported by established neuroanatomic and neuroinflammatory pathways. The choroid and uveal tract are innervated by branches of the trigeminal nerve, providing a direct substrate through which ocular inflammation may activate the trigeminovascular system (5). Inflammatory signaling within the choroid could promote peripheral sensitization of trigeminal afferents and secondarily drive central sensitization, producing headache phenotypes with overlapping features of migraine and TACs. The prominent autonomic features observed may similarly arise from trigeminal–autonomic reflex activation rather than indicating a primary hypothalamic disorder as in classic TACs.

Additional factors may further amplify this process. Systemic inflammatory or post-infectious states, including recent viral illness (e.g., COVID-19 infection in our patients), could enhance trigeminovascular excitability through mediators such as calcitonin gene-related peptide (CGRP), thereby lowering the threshold for headache generation in the context of ocular inflammation (6). Genetic polymorphisms involved in neuronal and hormonal signaling could also play a role in modulating susceptibility to BSCR-related headache, potentially contributing to inter-individual variability in headache phenotype and severity. However, these factors are best conceptualized as modulators of susceptibility rather than primary drivers, and temporality alone does not establish causality.

Taken together, these cases support a potential model in which BSCR-associated ocular inflammation acts as a peripheral generator of trigeminovascular activation, producing secondary headache phenotypes that mimic trigeminal autonomic cephalalgias. This framework reflects the underlying biology and aligns with ICHD-3 principles by prioritizing attribution to the active disease process rather than phenotypic resemblance.

## Conclusion

This is the first study to apply ICHD-3 criteria for diagnosing BSCR-associated headache. Our observations suggest that headache may represent a secondary manifestation of ocular inflammation in some patients with BSCR, with clinical features that can overlap with primary headache syndromes. However, given the small number of cases and the observational nature of this report, these findings should be interpreted with caution.

Research on the extraocular manifestations of BSCR remains limited, and current treatment strategies primarily target ophthalmologic symptoms. Future studies are needed to systematically characterize the prevalence, phenotypic spectrum, and temporal relationship of headache in BSCR and to clarify the underlying pathophysiological mechanisms linking ocular inflammation and trigeminovascular sensitization. Improved characterization may help distinguish secondary headache from coexisting primary headache disorders and inform more targeted treatment approaches.

## Data Availability

The original contributions presented in the study are included in the article/**Supplementary Material**. Further inquiries can be directed to the corresponding author.
