# Supplementary material for: Headache as an extraocular manifestation of birdshot chorioretinopathy: a case series
Source: Front Ophthalmol (Lausanne). 2026 Aug 13;6:1838200. doi: 10.3389/fopht.2026.1838200 (PMC13518146; doi:10.3389/fopht.2026.1838200)
Supplement: Supplementary file 1 [file Table1.pdf]

Supplemental Table 1. List of preventive and abortive medications and interventions trialed by Patient 1. These agents were either ineffective or caused adverse side effects. Headache severity ultimately decreased on a regimen of high-flow oxygen, cluster-dose galcanezumab (300 mg monthly), candesartan, tizanidine, and onabotulinumtoxinA.

Class	***Agent***
**Preventive**	
Angiotensin converting enzyme inhibitor	lisinopril
Anti-convulsant	topiramate<br>gabapentin
β-adrenergic receptor antagonist	propranolol
	metoprolol
Calcitonin gene-related peptide (CGRP) receptor antagonist	rimegepant
	atogepant
CGRP inhibitor, monoclonal antibody	erenumab<br>fremanezumab
Calcium channel antagonists	verapamil
	gabapentin
Carbonic anhydrase inhibitor	acetazolamide
Nerve block	trigeminal nerve blockade
	occipital nerve blockade
N-methyl-D-aspartate (NMDA) receptor antagonist	memantine
Non-steroidal anti-inflammatory drug (NSAID)	indomethacin, oral
Tricyclic antidepressant	amitriptyline
	protriptyline
**Abortive**	
--	high-flow oxygen
5-HT_3 receptor antagonist	ondansetron
Anti-convulsant	valproate, oral
CGRP receptor antagonist	ubrogepant
Corticosteroid	dexamethasone, intramuscular
Ergot alkaloid	dihydroergotamine, intramuscular<br>dihydroergotamine, nasal spray
$GABA_B$ receptor agonist	baclofen
Muscle relaxant	cyclobenzaprine
NSAID	ketorolac, intramuscular
Typical (first-generation) antipsychotic	prochlorperazine, oral<br>promethazine, oral
Triptan	sumatriptan, oral<br>sumatriptan, intramuscular<br>zolmitriptan nasal spray<br>eletriptan
	rizatriptan
Voltage-gated sodium channel inhibitor	lidocaine nasal spray<br>suzetrigine
